# SOCS1 Mimetic Peptide Suppresses Chronic Intraocular Inflammatory Disease (Uveitis)

**DOI:** 10.1155/2016/2939370

**Published:** 2016-09-15

**Authors:** Chang He, Cheng-Rong Yu, Mary J. Mattapallil, Lin Sun, Joseph Larkin III, Charles E. Egwuagu

**Affiliations:** ^1^Molecular Immunology Section, National Eye Institute, National Institutes of Health, Bethesda, MD 20892, USA; ^2^State Key Laboratory of Ophthalmology, Zhongshan Ophthalmic Center, Sun Yat-sen University, Guangzhou 510060, China; ^3^Immunoregulation Section, National Eye Institute, National Institutes of Health, Bethesda, MD 20892, USA; ^4^Department of Microbiology & Cell Science, University of Florida, Gainesville, FL 32611, USA

## Abstract

Uveitis is a potentially sight-threatening disease characterized by repeated cycles of remission and recurrent inflammation. The JAK/STAT pathway regulates the differentiation of pathogenic Th1 and Th17 cells that mediate uveitis. A SOCS1 mimetic peptide (SOCS1-KIR) that inhibits JAK2/STAT1 pathways has recently been shown to suppress experimental autoimmune uveitis (EAU). However, it is not clear whether SOCS1-KIR ameliorated uveitis by targeting JAK/STAT pathways of pathogenic lymphocytes or via inhibition of macrophages and antigen-presenting cells that also enter the retina during EAU. To further investigate mechanisms that mediate SOCS1-KIR effects and evaluate the efficacy of SOCS1-KIR as an investigational drug for chronic uveitis, we induced EAU in rats by adoptive transfer of uveitogenic T-cells and monitored disease progression and severity by slit-lamp microscopy, histology, and optical coherence tomography. Topical administration of SOCS1-KIR ameliorated acute and chronic posterior uveitis by inhibiting Th17 cells and the recruitment of inflammatory cells into retina while promoting expansion of IL-10-producing Tregs. We further show that SOCS1-KIR conferred protection of resident retinal cells that play critical role in vision from cytotoxic effects of inflammatory cytokines by downregulating proapoptotic genes. Thus, SOCS1-KIR suppresses uveitis and confers neuroprotective effects and might be exploited as a noninvasive treatment for chronic uveitis.

## 1. Introduction

Uveitis is a diverse group of potentially sight-threatening intraocular inflammatory diseases that accounts for more than 10% of severe visual handicaps in the United States. The disease may occur in the front of the eye (anterior uveitis), in the back of the eye (posterior uveitis), or throughout the eye (pan uveitis) and can be of infectious or autoimmune etiology [1]. Ocular pathology derives in part from sustained production of cytotoxic cytokines by inflammatory cells recruited into the optical axis during ocular inflammation. Current therapy for posterior or pan uveitis is systemic corticosteroid and longer therapy is required to prevent recurrence. However, ocular inflammation has responded poorly to corticosteroids alone and prolonged use of steroid in chronic uveitis is associated with serious side effects such as glaucoma or cataract and this has been a major impetus to develop less toxic and more specific therapies for uveitis [2].

Experimental autoimmune uveitis (EAU) shares essential pathological features with human uveitis and is the animal model of human pan uveitis. EAU has provided valuable insights into the pathophysiology of uveitis [3] and has been indispensable to the development and testing of new drugs or biologics for the treatment of uveitis [4, 5]. The experimental animal used in the majority of early studies on EAU had been the Lewis rat, an inbred strain that is highly susceptible to EAU induced by all known uveitogenic Ags. In contrast to rats, most strains of mice are resistant to EAU and the small number of mouse strains that are susceptible develops the disease only when immunized with the retinal Ags at doses much higher than those causing disease in Lewis rats. Studies on the EAU model suggest that uveitis is mediated in part by Th1 and Th17 cells and either T cell subset can independently induce EAU depending on the method of disease induction or physiological context [6–8]. In view of the inherent plasticity of mechanisms that regulate developmental pathways of T helper cells, therapeutic targeting of only one of these subsets could unexpectedly promote the expansion of the other. Thus, it is most expedient to develop strategies that would concurrently target both effector responses. A common feature shared by Th1 and Th17 is the obligatory requirement of STAT pathways for their development, and studies using mice deficient in STAT1 or STAT3 in the T cell compartment have revealed impairment of the development of Th1 and Th17 cells, respectively [9, 10]. In fact, the loss of STAT3 in T cells prevents the development of EAU in the mouse, suggesting that JAK/STAT pathways are potential therapeutic targets that can be exploited to mitigate uveitis [8]. Furthermore, unbridled activation of the JAK/STAT signaling pathways due to defective expression or silencing of suppressors of cytokine signaling 1 (SOCS1) or SOCS3 gene is also implicated in pathogenesis of a number of autoimmune diseases [11].

SOCS proteins are an eight-member family of cytokine-inducible negative feedback regulators that control the initiation, intensity, duration, and quality of cytokine responses [12, 13]. SOCS1 and SOCS3 are the best characterized members of the family and each possesses a kinase inhibitory region (KIR) that potently inhibits JAK/STAT signaling [11]. There is significant interest in developing strategies to efficiently deliver exogenous SOCS1 or SOCS3 into cells as potential therapeutic approach for the treatment of autoimmune diseases. However, a major impediment to therapeutic use of SOCS1 or SOCS3 is their relatively short half life and difficulty in developing methods to deliver the protein into cells [14]. In this study, we have synthesized a 16-amino acid peptide bearing a cell membrane-penetrating lipophilic group (SOCS1-KIR) that interacts with JAK2 autophosphorylation loop and inhibits JAK kinase activity [15]. We show here that topical administration of SOCS1-KIR confers protection from ocular pathology and mitigates chronic uveitis.

## 2. Materials and Methods

### 2.1. Animals

Lewis rats (6–8 weeks old) were purchased from Charles River Laboratory (Frederick, MD) and animal care and use was in compliance with National Institutes of Health (NIH) guidelines (Study numbers EY000262-19 and EY000372-14). The NIH Animal Care and Use Committee approved the protocol used in studies described in this paper (ASP number NEI-601).

### 2.2. Peptide Synthesis

An N terminal palmitoyl-lysine group was attached to SOCS1-KIR mimetic peptide (DTHFRTFRSHSDYRRI) and also to a SOCS1 scrambled peptide (KHRTDSRHSDRIYTFRF). Both peptides were synthesized to >95% purity at GenScript (Piscataway, NJ).

### 2.3. Induction of EAU and Histology

We induced EAU in age and sex matched Lewis rats by adoptive transfer of the R16 uveitogenic T cell line [16]. The R16 pathogenic T cell line is specific to peptide R16 (ADGSSWEGVGVVPDV, residues 1177–1191) of the bovine IRBP protein [16]. A total of 4 × 10^6^ R16 peptide-specific T cells were injected intraperitoneally (i.p.). For active immunization, the rats were immunized subcutaneously (s.c.) into both hind legs and back tail with a total volume of 200 *μ*L emulsion containing 25 *μ*g R16 peptide and CFA. On the day of disease induction and every day until day 9, rats were treated with 1 eye-drop/per eye (20 *µ*g in 10 *µ*L) of a scrambled 16-amino acid peptide (control peptide) or SOCS1-KIR and rats were sacrificed 12 days or 50 days after induction of EAU. Clinical disease was established and scored by slit-lamp microscopy examination and histology as described previously [17, 18]. The clinical disease was graded and scored on a scale from 0 (no disease) to 4 (severe disease) based on inflammatory signs in the anterior chamber [17]. Eyes for histological EAU evaluation were harvested, fixed immediately with 4% glutaraldehyde (Thermo Fisher Scientific, Rockville, MD, USA) in phosphate-buffered saline (PBS) for 1 hour, and followed by fixation in 10% buffered formaldehyde (Sigma-Aldrich Corp.) for 24 hours. They were then transferred in 70% alcohol and stored at 4°C until embedded in paraffin. Paraffin embedded eyes was then serially sectioned in the vertical pupillary-optic nerve plane. All sections were stained with hematoxylin and eosin and evaluated in a blinded fashion. For histopathologic evaluation, the iris-ciliary body complex, anterior chamber, vitreous, and retina were observed under a light microscope. Based on the number and extent of lesions seen in the HE-staining section, histological scores were provided on a scale of 0 to 4 using criteria described previously [17, 19].

### 2.4. Imaging Rat Anterior Segment by Digital Slit-Lamp Microscopy System

Ocular anterior segment examinations were performed using a digital slit-lamp system (Carl Zeiss). Briefly, following systemic administration of systemic anesthesia [intraperitoneal injection of ketamine (1.4 mg/rat) and xylazine (0.12 mg/rat)], the rats were observed under the slit-lamp microscopy and the images were captured at various time points after adoptive transfer of uveitogenic T cells. To avoid a subjective bias, evaluation of the anterior segment photographs was conducted without knowledge of the rat's identity by a masked observer. At least two images (1 by diffuse illumination, 1 by fissure illumination) were taken from each eye. The clinical grading system for ocular inflammation was as previously established [19].

Severity of EAU was scored (range, 0 to 4) using slit-lamp microscope by an ophthalmologist blinded to the experimental groups. Scoring started on the day of immunization (day 1), continuing every other day until the duration of the study. Grade 0 represents no disease (eyes reflected light [red reflex] and were translucent); grade 0.5 (trace), dilated blood vessels in the iris; grade 1, enlargement of the iris vessel and abnormal pupil contraction; grade 2, cellular infiltrates and hazy anterior chamber with decreased red reflex; grade 3, moderately opaque anterior chambers with visible pupils and dull red reflex; grade 4, opaque anterior chambers with obscured pupils and absence of red reflex. Grading was as previously established [17, 19].

### 2.5. Cross-Sectional Imaging of Rat Anterior Segment by Optical Coherence Tomography (OCT)

Optical coherence tomography (OCT) is a noninvasive procedure that allows visualization of internal microstructure of various eye structures in living animals. An SD-OCT system with 1180 nm center wavelength broadband light source (Bioptigen, NC) was used for* in vivo* noncontact imaging of eyes from scrambled peptide or SOCS1-KIR rats. Before OCT imaging was performed, each rat was anesthetized. The anesthetized rat was immobilized using adjustable holder that could be rotated easily allowing for horizontal or vertical scanning. Each scan was performed at least twice, with realignment each time. The dimension of the scan (in depth and transverse extent) was adjusted until the optimal signal intensity and contrast were achieved. Retinal thickness was measured from the central retinal area of all images obtained from both horizontal and vertical scans from the same eye, using the system software, and averaged.

### 2.6. Analysis of Inflammatory Cells by Flow Cytometry

Cells were isolated from the retina, spleen, or draining lymph nodes (LN) as described [8] and cell surface protein expression was detected and quantified by FACS (fluorescence-activated cell sorting). For intracellular cytokine detection, cells were restimulated for 5 h with PMA (20 ng/mL)/ionomycin (1 *µ*M). Golgi-stop was added in the last hour and intracellular cytokine staining was performed using BD Biosciences Cytofix/Cytoperm kit as recommended (BD Pharmingen, San Diego, CA). FACS analysis was performed on a Becton-Dickinson FACSCalibur (BD Biosciences) using Ag-specific monoclonal antibodies and corresponding isotype control Abs (PharMingen, San Diego, CA) as described [6]. The rat-specific mAbs used were from BD (IL-10, IFN-*γ*, CD3, CD8, CD4, and CD11b/c) or E-Bioscience (IL-17A).

### 2.7. Lymphocyte Proliferation Assay

Cells isolated from the lymph nodes or spleens were restimulated* in vitro* with the R16 peptide in the presence of the scrambled peptide or SOCS1-KIR. After 48 h, cultures were pulsed with ^3^H-thymidine (0.5 *µ*Ci/10 *µ*L/well) for 12 additional hours and analyzed as described [5]. The presented data are the mean CPM ± SEM of the responses of five replicate cultures.

### 2.8. Quantitative (qPCR) Analysis

Total RNA was extracted from lymphocytes and retinal cells using the TRIzol reagent according to the procedures recommended by the manufacturer (Life Technologies, Gaithersburg, MD). All RNA samples were digested with RNase-free DNase 1 (Life Technologies) for 30 minutes, purified by phenol/chloroform extractions, and precipitated in 0.4 M LiCl. RNA (10 *µ*g), a commercial synthesis system (SuperScript III Reverse Transcriptase; Life Technologies), and oligo(dT) were used for first-strand cDNA synthesis as previously described. First-strand synthesis containing each mRNA sample but without reverse transcriptase was performed to control for possible DNA contamination; failure to obtain RT-PCR products with any of the PCR amplimers confirmed the absence of contaminating DNA. All cDNA preparations used were suitable for PCR amplification on the basis of efficient amplification of a *β*-actin sequence. Real-time PCR was performed on a fast real-time PCR system (ABI 7500) and PCR parameters were as recommended by the manufacturer (TaqMan Universal PCR Kit; Applied Biosystems). Primers and probes used were purchased from Applied Biosystems. The mRNA expression levels were normalized to the levels of GAPDH housekeeping gene.

### 2.9. Cytotoxic Assay for Detection of Apoptosis in Retinal and RPE Cells

Apoptosis was induced in retinal or RPE cells cultured for 1 and 3 hours in medium containing 1 *µ*M staurosporine (Sigma) or 24 hours in medium containing IL-17 (50 ng/mL), SOCS1-KIR, or scrambled peptide. Cell death was assessed by FACS analysis of Annexin-V- or 7-AAD- (7-amino-actimycin D-) labeled cells according to the manufacturer (BD Biosciences). Mouse photoreceptor cell line (661 W), human retinal pigment epithelial cell line (ARPE-19), and human Muller cell line (MI0-M1) were used in this study.

### 2.10. Statistical Analysis

Statistical analyses were performed by independent two-tailed Student's *t*-test. For EAU scores, nonparametric Mann-Whitney method was used. Probability values of ≤0.05 were considered statistically significant. The data are presented as mean ± SEM.

## 3. Results

### 3.1. SOCS1-KIR Reduced the Severity of Acute and Chronic Uveitis

**Figure 1:**
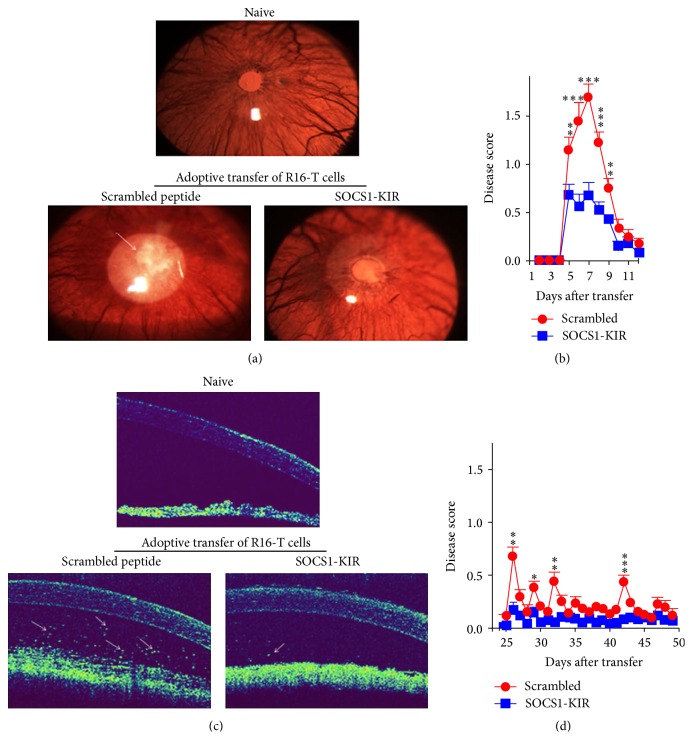
SOCS1-KIR suppressed inflammation in the anterior segment of the eye during EAU. (a) We induced EAU by adoptive transfer of uveitogenic R16-specific T cells and the rats were treated with SOCS1-KIR or scrambled peptide (control). Representative images of the anterior eye segment were taken 6 days after EAU induction using a slit-lamp microscopy system. The images reveal marked inflammation with hazy anterior chamber and obscured pupil in the control rats compared to rats treated with SOCS1-KIR. (b) Clinical scores of the eyes at various time points after induction of EAU. (c) Visualization of longitudinal structure of the anterior eye segment by anterior segment OCT. Representative OCT images taken 8 days after disease induction show markedly increased inflammatory cells (white arrows) in the anterior chamber of rats treated with scrambled peptide compared to rats treated with SOCS1-KIR. (d) Clinical scores of the eyes at day 50 after induction of EAU. Clinical scores and assessment of disease severity were based on changes at the iris vessels and anterior chamber as described in the text. Data presented are the average of six rats per group and results expressed as the mean ± SEM. ^*∗*^ *P* < 0.05, ^*∗∗*^ *P* < 0.005, ^*∗∗∗*^ *P* < 0.001.

In a previous study, we showed that a suppressor of cytokine signaling 1 mimetic peptide (SOCS1-KIR) that inhibits JAK2/STAT1 pathway suppressed EAU in mice [20]. However, SOCS1-KIR also targets JAK/STAT pathways of most inflammatory cells and it is not clear whether the suppression of EAU derived from inhibition of macrophages and antigen-presenting cells that enter the retina following induction of EAU. We induced EAU by adoptive transfer of the rat R16 uveitogenic T cell line. In contrast to EAU induced by active immunization, adoptive transfer circumvents the confounding effects of antigen-presenting cells as pathogenic lymphocytes are directly delivered to the mice without the agency of antigen-presenting cells. On the day of adoptive transfer and every day until day 9 after adoptive transfer, rats were treated with 1 eye-drop/per eye (20 *µ*g in 10 *µ*L) of a scrambled 16-amino acid peptide (control peptide) or SOCS1-KIR. The peptide was administrated at a relatively low dose by local eye-drops compared with systemic administration whereby only a fraction of dose delivered is expected to reach the retina due to the blood-retinal barrier. Besides, the peptide was administrated in a noninvasive way avoiding the traumatic and infected risk. This experimental approach ensured direct delivery of the drug to the target site and mitigated degradation or clearance of the drug before reaching its target tissue. Disease progression was monitored by slit-lamp microscopy or optical coherent tomography (OCT), beginning from day 6 following induction of EAU. Unlike EAU in most mouse strains, it is of note that rats are highly susceptible to EAU and often present with pan uveitis with very severe anterior inflammation that impede observation of posterior uveitis by fundoscopy. Thus, we used slit-lamp microscopy to score the disease severity. Slit-lamp images of ocular anterior segment showed severe inflammation with hazy anterior chamber and obscured pupil in rats received control peptide (Figure 1(a)). In contrast, clinical scores of EAU were significantly reduced in the eyes of rats that were treated with the SOCS1-KIR mimetic compared to the control peptide (Figures 1(a) and 1(b)). Analysis of the eyes by OCT revealed a substantial decrease in the number of inflammatory cells in anterior chamber of the eyes of the SOCS1-KIR-treated rats (Figure 1(c)), confirming the suppressive effects of the SOCS1-KIR mimetic peptide. We also examined the effects of SOCS1-KIR on chronic uveitis by analyzing slit-lamp. Some reports suggest that relapse of EAU in rats by R16 uveitogenic T cells might begin as early as day 16 or day 25 after adoptive transfer [21–23]. We therefore monitored uveitis in the rat eyes until 50 days after induction of EAU and the rats treated with SOCS1-KIR were found to develop significant lower EAU scores compared to rats treated with control peptides (Figure 1(d)). Taken together, these results suggest that SOCS1 mimetic peptide may be effective for treating both acute and chronic uveitis.

### 3.2. SOCS1-KIR Mitigates Retinitis, Iridocyclitis, and Pan Uveitis

**Figure 2:**
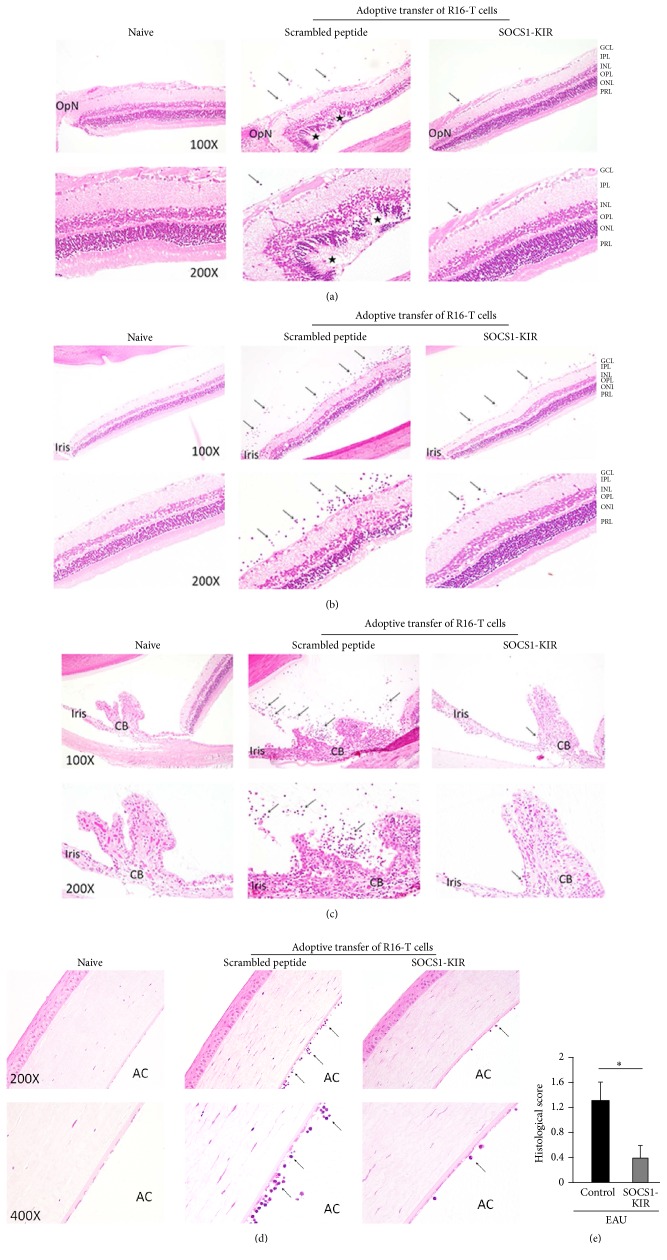
SOCS1-KIR conferred protection from posterior uveitis and pan uveitis. (a–d) We induced EAU in rats by adoptive transfer of uveitogenic R16 T cell line and the rats were treated with either SOCS1-KIR or scrambled peptide (control). The eyes were analyzed by histology on day 12 after disease induction. Representative results of histological analyses of the central retina (a), peripheral retina (b), iris and ciliary body (c), and the cornea (d) are shown. Images reveal substantial reduction of the numbers of inflammatory cells (arrows) in the vitreous and other tissues of rats treated with SOCS1-KIR compared to the scrambled peptide. Hallmark features of severe uveitis such as retinal folds and photoreceptor cell loss (asterisks), infiltrated inflammatory cells (arrows), and disorganized structure of retinae are markedly alleviated in eyes of rats treated with SOCS1-KIR. The sections were stained with hematoxylin and eosin. GCL, ganglion cell layer; IPL, inner plexiform layer; INL, inner nuclear layer; OPL, outer plexiform layer; ONL, outer nuclear layer; PRL, photoreceptor layer; OpN, optic nerve; CB, ciliary body; AC, anterior chamber. (e) Histological score and assessment of disease severity based on changes of the retina architecture, retinal vessels, and presence of retinal and choroidal infiltrates. Results represent at least three independent experiments. ^*∗*^ *P* < 0.05.

Uveitis can occur in the front of the eye (anterior uveitis), in the back of the eye (posterior uveitis), or throughout the eye (pan uveitis) [1]. We therefore examined whether topical treatment of the rats with the SOCS1-KIR mimetic peptide would be effective in suppressing EAU pathology in various areas of the eye, including the anterior and posterior ocular segments. Images provided are from representative anatomical landmarks, such as optic nerve, iris, ciliary body, or the cornea. Analysis of eyes of rats treated with the SOCS1-KIR or control scrambled peptide 12 days after adoptive transfer of pathogenic R16 T cells by histology shows the development of pan uveitis in the rats treated with the scrambled peptide. Histological data on the eyes of untreated mice with EAU did not reveal any nonspecific effect of scrambled peptide on inflammation. These results contrast with remarkable decrease in inflammatory cells in the central retina (Figure 2(a)), peripheral retina (Figure 2(b)), iris/ciliary body (Figure 2(c)), or cornea (Figure 2(d)) of the rats treated with SOCS1-KIR and reduction in the levels of inflammatory cells in these tissues correlated with reduced ocular pathology as indicated by slit-lamp microscopy (Figures 1(a) and 1(b)) and OCT (Figure 1(c)). Histological scores for pathological changes and inflammation in the peripheral and central retina revealed significant lower EAU and disease scores were graded as previously reported [17, 18].

### 3.3. SOCS1-KIR Suppressed the Levels of Inflammatory Cells in the Retina during EAU

**Figure 3:**
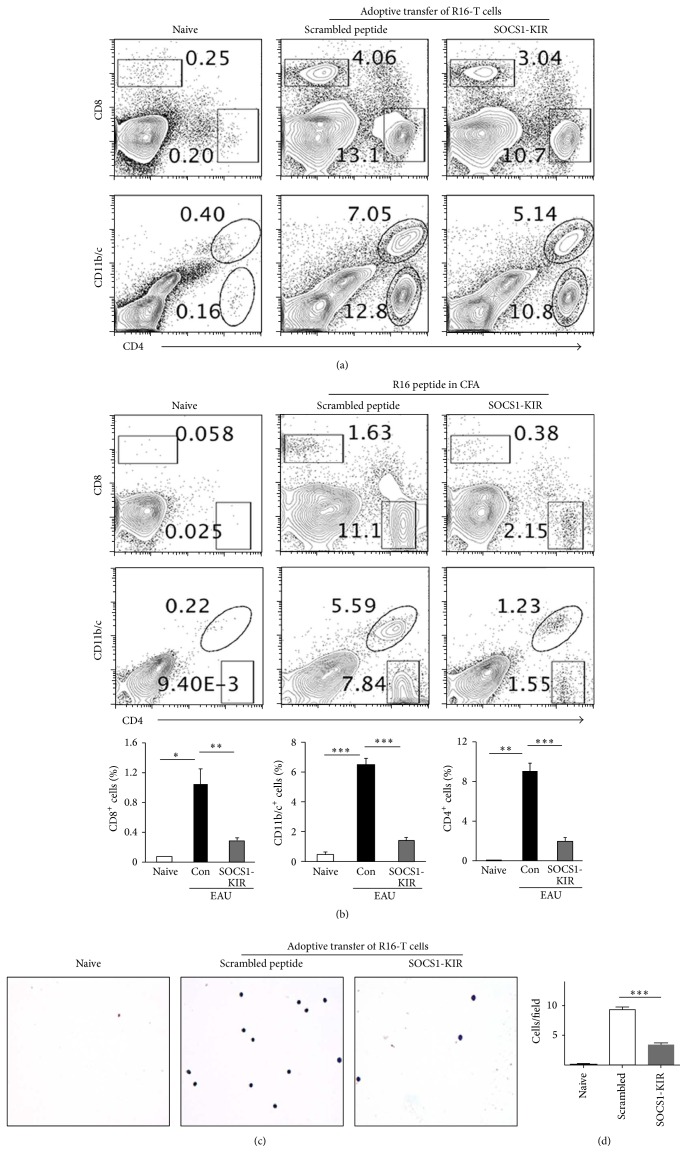
SOCS1-KIR suppressed the infiltration of inflammatory cells into the retina during EAU. We induced EAU in rats by adoptive transfer of uveitogenic R16 T cell line (a, c, d) or by active immunization with R16 peptide (b) and the rats were treated with SOCS1-KIR or scrambled peptide (control). Inflammatory cells present in the retina on day 12 after disease induction were analyzed by flow cytometry. Numbers in quadrants indicate percentage of T lymphocytes (CD4^+^, CD8^+^) or myeloid cells (CD11b/c^+^) in the retinae. (c, d) Ocular aqueous and vitreous humor of rats were collected 50 days after adoptive transfer. We smeared 1 mL ocular fluid from each rat eye on the glass slide and stained for cytological analysis with giemsa stain (c) and the cells were then quantified (d). Results represent at least three independent experiments. ^*∗*^ *P* < 0.05, ^*∗∗*^ *P* < 0.005, and ^*∗∗∗*^ *P* < 0.001.

Microglial cells have recently been shown to contribute to experimental autoimmune encephalomyelitis (EAE) by facilitating the expansion and effector functions of Th1 and Th17 cells [24]. Because this animal model of multiple sclerosis (MS) shares essential immunopathological features with EAU, we examined whether the suppression of EAU in rats treated with SOCS1-KIR derived in part from inhibiting expansion of inflammatory cells in the retina during EAU. We induced EAU by adoptive transfer of the R16 pathogenic T cells (Figures 3(a), 3(c), and 3(d)) or by active immunization with the R16 peptide in CFA (Figure 3(b)) and the rats received either SOCS1-KIR or control peptide. On day 12 after disease induction the eyes were harvested and after digestion with collagenase we isolated the inflammatory cells in the retina. We then analyzed by flow cytometry the levels of cells expressing CD4, CD8, CD11b, and CD11c that are cell surface protein markers of T cells, microglia, and other myeloid cells. These studies revealed reductions in the levels of lymphoid and myeloid cells in the eyes of rats treated with SOCS1-KIR (Figures 3(a) and 3(b)). We also collected ocular aqueous and vitreous humor from the rats 50 days after adoptive transfer and stained the cells with giemsa stain and cytological analysis revealed marked reductions in inflammatory cells in both the anterior and the posterior chambers of the eye (Figures 3(c) and 3(d)). Taken together, these results suggest that SOCS1-KIR mitigated uveitis, in part, by suppressing the level of inflammatory cells in the retina during EAU.

### 3.4. SOCS1-KIR Inhibited Expression of Inflammatory Cytokines and Chemokines during EAU

**Figure 4:**
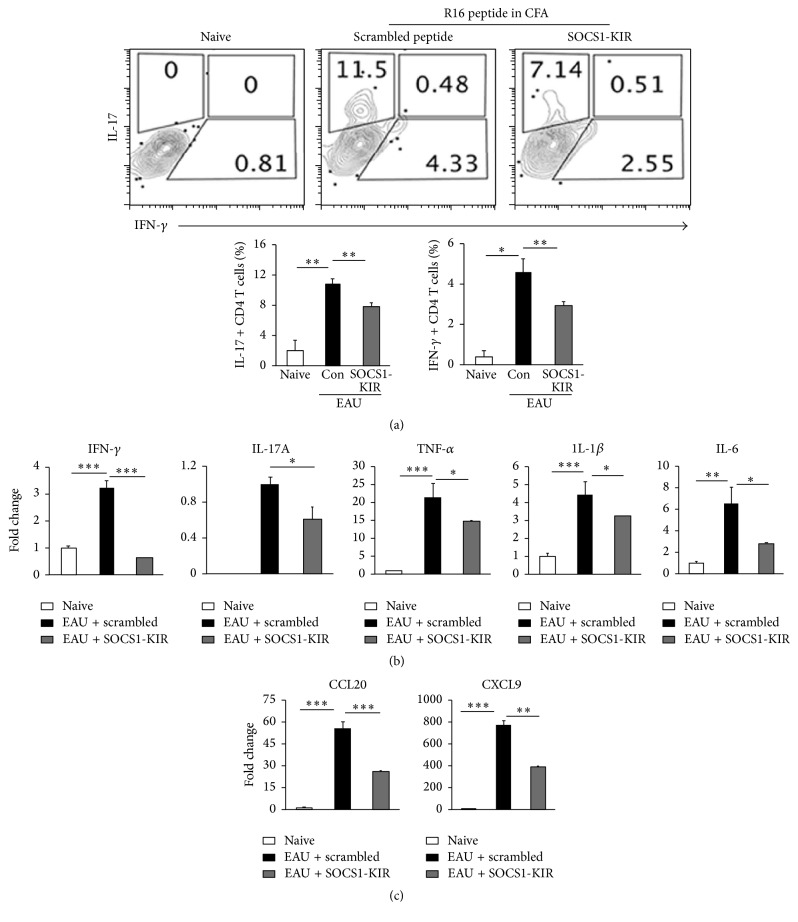
SOCS1-KIR inhibited expression of proinflammatory cytokines and chemokines during EAU. We induced EAU in rats by active immunization with R16 peptide and the rats were treated with SOCS1-KIR or scrambled peptide (control). (a) Inflammatory cells present in the retina 12 days after disease induction were analyzed by flow cytometry. Plots were gated on CD4^+^ T cells and numbers in quadrants indicate percentage of CD4^+^ T cells expressing IL-17 or IFN-*γ*. (b, c) qRT-PCR analysis of genes coding for inflammatory cytokines and chemokines in the retinae. Results represent at least three independent experiments. ^*∗*^ *P* < 0.05, ^*∗∗*^ *P* < 0.005, ^*∗∗∗*^ *P* < 0.001.

**Figure 5:**
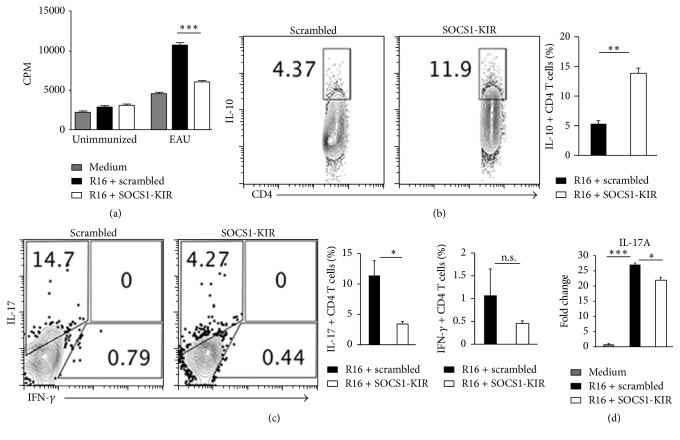
SOCS1-KIR induced expansion of IL-10-producing T cells while suppressing Th17 cells. We isolated cells from the spleen and LN of rats with EAU, restimulated the cells* in vitro* with the R16 peptide in medium containing SOCS1-KIR or scrambled peptide, and then analyzed the cells by the thymidine incorporation assay (a), intracellular cytokine staining assay (b, c), or qPCR (d). Proliferative responses were analyzed in six replicate cultures and data presented as CPM indicate the mean values of six replicate cultures. For intracellular staining, the cells were gated on CD3^+^ T cells and analysis was on CD4^+^ T cells. Numbers in quadrants (b, c) indicate percentage of IL-10-, IL-17-, or IFN-*γ*-expressing CD4^+^ T cells. Results represent three independent experiments. ^*∗*^ *P* < 0.05, ^*∗∗*^ *P* < 0.005, ^*∗∗∗*^ *P* < 0.001 (Student's two-tailed *t*-test).

We next induced EAU in the rats by active immunization with R16/CFA and examined directly the effects of SOCS1-KIR treatment on the recruitment of Th1 and Th17 cells into the retina. We show here a 41% and 38% reduction in the levels of Th1 and Th17 subsets, respectively, in the retinae of rats treated with SOCS1-KIR compared to control rats treated with the scrambled peptide (Figure 4(a)). We also analyzed the levels of proinflammatory cytokines and chemokines in the retina by qPCR. Consistent with reduced inflammation in the retina of rats treated with the SOCS1-KIR mimetic peptide, the expression of proinflammatory cytokines such as IFN-*γ*, IL-17A, TNF-*α*, IL-1*β*, and IL-6 was markedly decreased in the retinae of SOCS1-KIR-treated rats (Figure 4(b)). Moreover, the downregulation of the proinflammatory genes correlated with decrease in mRNA transcripts of CCL20 (MIP3*α*) and CXCL9 (MIG), two chemokines that mediate the recruitment of inflammatory cells, especially Th17 cells and Th1 cells, to sites of inflammation (Figure 4(c)). Cells isolated from the spleen and lymph nodes of the rats were restimulated* in vitro* with the R16 peptide in medium containing the scrambled peptide or SOCS1-KIR. Analysis of the cells by the thymidine incorporation assay shows that the proliferative response to R16 peptide was significantly inhibited by SOCS1-KIR (Figure 5(a)), indicating that targeting the JAK/STAT pathway can be effective in suppressing the expansion of uveitogenic T cells. Intracellular cytokine staining (Figures 5(b) and 5(c)) and qPCR (Figure 5(d)) analysis further revealed that the inhibitory effects of SOCS1-KIR correlated with the expansion of IL-10-producing regulatory T cells (Figure 5(b)) and the diminution of Th17 level (Figure 5(c)). Together, these results suggest that SOCS1-KIR may confer protection from EAU by regulating the transcription of anti-inflammatory and proinflammatory cytokine genes and the decrease in the percentage of cells expressing chemokine receptors associated with Th1 and Th17 subset underscores the lower levels of these cells in the retina of SOCS1-KIR-treated rats.

### 3.5. SOCS1-KIR Suppressed EAU Pathology by Protecting Resident Retinal Cells from Apoptosis

**Figure 6:**
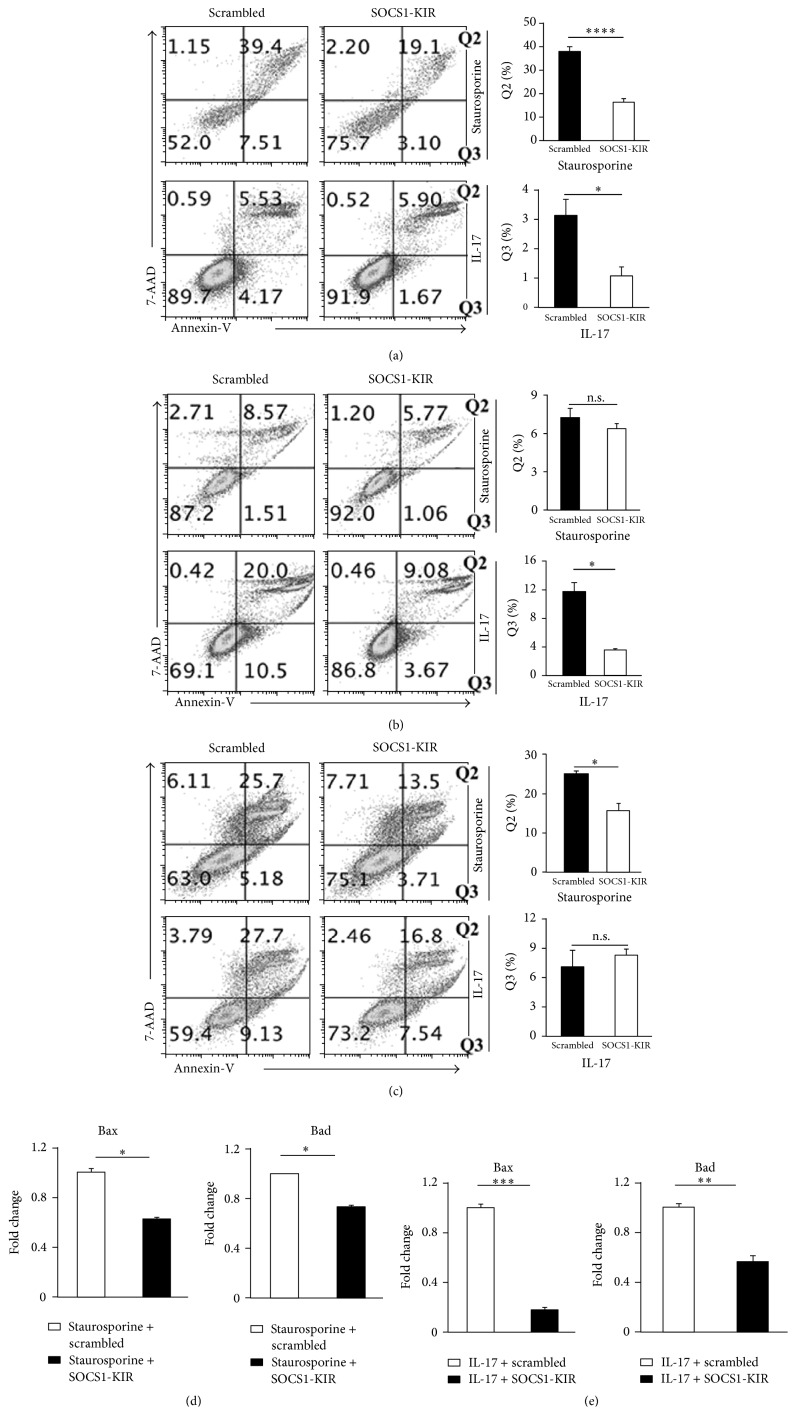
SOCS1-KIR mitigated EAU pathology by protecting resident retinal cells from apoptosis retinal photoreceptor cells (a), RPE cells (b), or Muller cells (c) were cultured in medium containing the proapoptotic reagent, staurosporine (2 *µ*M), IL-17 (50 ng/mL), SOCS1-KIR, or the scrambled peptide. We determined the percentage of the cells undergoing apoptosis or necrosis by the Annexin-V staining assay. (d, e) The photoreceptor cells were also analyzed for the expression of proapoptotic genes by qPCR. Results represent at least three independent experiments. ^*∗*^ *P* < 0.05, ^*∗∗*^ *P* < 0.005, ^*∗∗∗*^ *P* < 0.001, and ^*∗∗∗∗*^ *P* < 0.0001 (Student's *t*-test (two-tailed)).

Retinal pathology in EAU derives to a large extent from cytotoxic effects of proinflammatory cytokines secreted by inflammatory cells that infiltrate the retina. Macrophages and microglial cells are thought to locally amplify the responses of uveitogenic T cells and thereby induce the destruction of photoreceptors and retinal tissue. However, the retina is also thought to protect itself from cytokine-mediated cytotoxicity by activating endogenous antiapoptotic mechanisms inherent in resident retinal cells. We, therefore, examined whether SOCS1-KIR might mitigate ocular pathology during EAU by protecting the resident retinal cells from apoptosis. Photoreceptor, Muller, and retinal pigment epithelial cells are potential targets susceptible to inflammatory insults during intraocular inflammation. To address this issue, we used the apoptosis-inducing chemical, staurosporine, to induce apoptosis* in vitro* in two well-characterized resident retinal cell lines [(photoreceptor cell line (661 W), human Muller cell line (MI0-M1) photoreceptor, and Muller cells)] as well as the retinal pigment epithelial cells (ARPE-19) that play critical roles in protecting the retina from inflammatory insults. Apoptotic and necrotic cells were assessed by Annexin-V and 7-AAD staining assays, respectively. We show here that photoreceptor cells (Figure 6(a)), RPE cells (Figure 6(b)), or Muller cells (Figure 6(c)) cultured in medium containing staurosporine were partially protected from apoptosis when treated with SOCS1-KIR compared to cells treated with the scrambled peptide. IL-17, the critical pathogenic cytokine in EAU, is also thought to exert cytotoxic effects. Retinal cells or RPE cells were cultured in medium containing recombinant IL-17. Again, cells treated with SOCS1-KIR exhibited enhanced survival over those treated with the scrambled peptide (Figures 6(a)–6(c)), providing further evidence that SOCS1-KIR might protect resident ocular cells from apoptosis. In addition, the resistance to apoptosis in the cells treated with SOCS1-KIR correlated with decreased expression of proapoptotic genes (Figures 6(d) and 6(e)).

### 3.6. SOCS1-KIR Does Not Inhibit Peripheral Immune Responses

**Figure 7:**
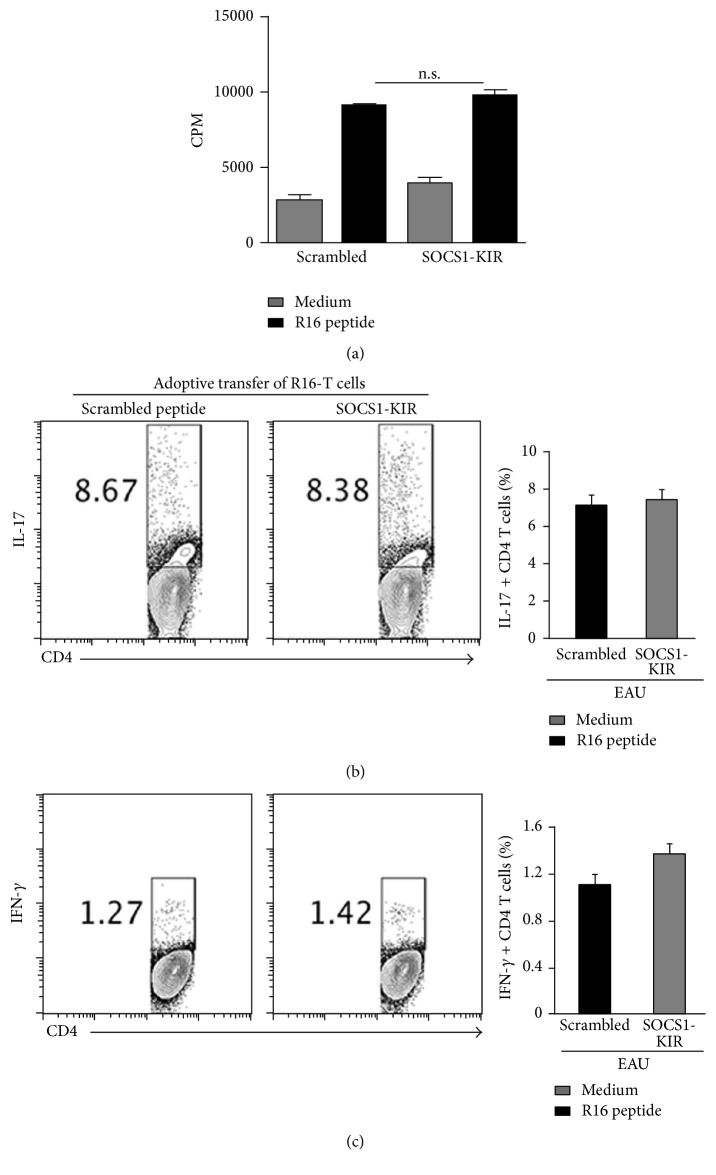
SOCS1-KIR has no effects on systemic immune response. We induced EAU by adoptive transfer of pathogenic R16 T cells and the rats were treated by topical administration of SOCS1-KIR or scrambled peptides as described above. Spleen cells from the rats were restimulated* in vitro* with indicated peptide and analyzed by the thymidine incorporation (a) or intracellular cytokine staining assay (b, c). Proliferative responses were analyzed in six replicate cultures and data presented as CPM (a). Numbers in quadrants indicate percentage of CD4^+^ T cells expressing IL-17 or IFN-*γ* (b, c). *P* = n.s. (No significance) > 0.05 (Student's two-tailed *t*-test).

SOCS1-KIR inhibits responses in the retina (Figure 1). It was, therefore, of interest to know whether its immune suppressive functions extend to the peripheral immune system or mainly localized in the eye. We induced EAU by adoptive transfer of pathogenic R16 T cells and rats were treated with topical administration of SOCS1-KIR or scrambled peptides as described above. Twelve days after EAU induction cells were isolated from the spleen or lymph nodes of the rats and restimulated with the medium alone or the R16 peptide. Proliferative responses of quadruplet cultures revealed no significant differences between cells from rats treated with the scrambled peptides and the rats that received the SOCS1-KIR mimetic peptide (Figure 7(a)). Intracellular cytokine analysis also indicated equivalent levels of IFN-*γ*-producing Th1 and IL-17-producing Th17 cells from rats treated with the scrambled peptides and those that received the SOCS1-KIR mimetic peptide (Figure 7(b)). These results suggest that localized administration SOCS1-KIR in the eye had no effects on effector lymphocyte responses in the spleen or LN. Thus, while topical administration of SOCS1-KIR is effective therapy for suppressing inflammatory responses in the eye, it does not pose danger of inducing generalized immune suppression of the peripheral immune system.

## 4. Discussion

The repeated cycles of remission/recurrent inflammation that characterize chronic uveitis necessitates targeting the autoreactive T cells and their autoimmune responses that mediate disease recurrence. In previous reports, we had shown that expression of SOCS1 and SOCS3 increases in the retina during EAU, suggesting that the induction of these SOCS proteins during ocular inflammation might suppress immune responses by regulating the intensity and duration of proinflammatory cytokine signals [25–27]. Defective expression of SOCS1 in patients with scleritis, taken together with studies showing that rats with targeted overexpression of SOCS1 in the retina are protected from uveitis [28], implies that administration of SOCS1 mimetic peptides may be useful in treating uveitis. In this study, we have used SOCS1-KIR, a 16-amino acid lipophilic SOCS1 peptide, to suppress chronic pan uveitis in the rat, a species unlike the mouse, that manifests chronic relapsing uveitis. It is important to note that SOCS1-KIR targets JAK1 and JAK2, suggesting that it potentially inhibits pathways regulated by SOCS3.

While systemic administration of biologics such as daclizumab is effective in several inflammatory diseases including arthritis and atopic dermatitis, pharmacokinetics considerations have raised doubts of their efficacy in the neuroretina, a tissue that is sequestered from peripheral immune system by the blood retina barrier [9, 29]. In addition, large systemic doses of the drugs are often required to achieve therapeutic concentrations in the retina and this can induce adverse side effects. This was the impetus for examining whether topical delivery of SOCS1-KIR would be effective. We selected to treat the rats by topical treatment of the SOCS1-KIR because it allowed the direct delivery of the drug to the ocular tissue. The advantage of this approach is that it ensures less degradation or clearance of the drug before reaching its target tissue as well as low systemic absorption. The insignificant level of systemic absorption of the peptide after topical administration is indicated by our data showing that SOCS1-KIR topical treatment did not exert any inhibitory effects on peripheral immune responses. Of clinical importance, topical administration of SOCS1-KIR induced sustained therapeutic suppression of uveitis 50 days after induction of EAU and this method of drug administration did not cause any apparent discomfort to the mice. It is, therefore, of interest to determine whether the observed efficacy of a single eye-drop of SOCS1-KIR per day pertains to other inflammatory conditions of the eye, particularly in suppressing postoperative inflammation or cystoid macular edema. Eye-drops administration would also obviate adverse side effects associated with invasive therapeutic approaches, such as intravitreal injection of biologics, which can induce high intraocular pressure, endophthalmitis, retinal detachment, haemorrhage, and cataracts. Although we have not presented data on the effects of SOCS1-KIR on cells from patients with uveitis, the data presented here suggest that topical administration of SOCS1-KIR should be investigated as potential noninvasive effective therapy for mitigating human uveitis and other ocular inflammatory diseases.

The JAK/STAT pathway is one of the most important pathways utilized by cells of the innate and adaptive immune systems to initiate and regulate immune responses [9]. The accumulated knowledge on the role STAT proteins in regulating the differentiation, growth, and effector functions of inflammatory cells begs for translation of this knowledge to therapy in inflammatory and autoimmune diseases. Although many STAT inhibitors have been developed, relatively few have been tested or assessed as therapeutic agents in animal models of human diseases. In a previous study, we generated membrane-penetrating SOCS1 protein (MTS-SOCS1) and showed that it inhibits STAT pathway and suppresses expansion of the uveitogenic Th17 cells* in vitro* [30]. However, it could not suppress uveitis in mice due to its rapid clearance [30]. Here, our results suggest that topical SOCS1-KIR administration could ameliorate uveitis pathology on two distinct levels: (1) through direct inhibition of immunopathology mediated by uveitogenic T lymphocytes and (2) by protecting resident ocular cells from apoptosis during chronic intraocular inflammatory diseases. The use of the SOCS1-KIR mimetic peptide to suppress acute as well as relapsing uveitis in this study thus represents an important addition to the armamentarium of remedies for several of the potentially blinding chronic uveitides. It is, therefore, of note that the kinase inhibitory region of SOCS1 has also been shown to inhibit acute EAU as well as experimental allergic encephalomyelitis [15, 20]. In comparison to some commonly used biologics, the lipophilic SOCS1-KIR peptide without the SOCS box, which significantly curtails the half-life of SOCS protein, has the advantage of sustained* in vivo* inhibitory activity. We envision potential use of SOCS1-KIR in combination with steroids in context of a corticosteroid-sparing immunosuppressive therapy.

## References

[1] Nussenblatt R. B. (1990). The natural history of uveitis. *International Ophthalmology*.

[2] Nussenblatt R. B. (2002). Bench to bedside: new approaches to the immunotherapy of uveitic disease. *International Reviews of Immunology*.

[3] Caspi R. R. (2010). A look at autoimmunity and inflammation in the eye. *The Journal of Clinical Investigation*.

[4] Wang R.-X., Yu C.-R., Mahdi R. M., Egwuagu C. E. (2012). Novel IL27p28/IL12p40 cytokine suppressed experimental autoimmune uveitis by inhibiting autoreactive Th1/Th17 cells and promoting expansion of regulatory T cells. *Journal of Biological Chemistry*.

[5] Wang R.-X., Yu C.-R., Dambuza I. M. (2014). Interleukin-35 induces regulatory B cells that suppress autoimmune disease. *Nature Medicine*.

[6] Amadi-Obi A., Yu C.-R., Liu X. (2007). T_H_17 cells contribute to uveitis and scleritis and are expanded by IL-2 and inhibited by IL-27/STAT1. *Nature Medicine*.

[7] Luger D., Silver P. B., Tang J. (2008). Either a Th17 or a Th1 effector response can drive autoimmunity: conditions of disease induction affect dominant effector category. *The Journal of Experimental Medicine*.

[8] Liu X., Yun S. L., Yu C.-R., Egwuagu C. E. (2008). Loss of STAT3 in CD4+ T cells prevents development of experimental autoimmune diseases. *Journal of Immunology*.

[9] Egwuagu C. E., Larkin J. (2013). Therapeutic targeting of STAT pathways in CNS autoimmune diseases. *JAK-STAT*.

[10] Egwuagu C. E. (2009). STAT3 in CD4+ T helper cell differentiation and inflammatory diseases. *Cytokine*.

[11] Alexander W. S., Hilton D. J. (2004). The role of Suppressors of Cytokine Signaling (SOCS) proteins in regulation of the immune response. *Annual Review of Immunology*.

[12] Hilton D. J. (1999). Negative regulators of cytokine signal transduction. *Cellular and Molecular Life Sciences*.

[13] Yasukawa H., Sasaki A., Yoshimura A. (2000). Negative regulation of cytokine signaling pathways. *Annual Review of Immunology*.

[14] Jo D., Liu D., Yao S., Collins R. D., Hawiger J. (2005). Intracellular protein therapy with SOCS3 inhibits inflammation and apoptosis. *Nature Medicine*.

[15] Jager L. D., Dabelic R., Waiboci L. W. (2011). The kinase inhibitory region of SOCS-1 is sufficient to inhibit T-helper 17 and other immune functions in experimental allergic encephalomyelitis. *Journal of Neuroimmunology*.

[16] Caspi R. R., Silver P. B., Chan C.-C. (1996). Genetic susceptibility to experimental autoimmune uveoretinitis in the rat is associated with an elevated Th1 response. *The Journal of Immunology*.

[17] Caspi R. R., Coligan J. E., Kruisbeek A. M., Margulies D. H., Shevech E. M., Strober W. (2003). Experimental autoimmune uveoretinitis in the rat and mouse. *Current Protocols in Immunology*.

[18] Agarwal R. K., Silver P. B., Caspi R. R. (2012). Rodent models of experimental autoimmune uveitis. *Methods in Molecular Biology*.

[19] Yadav U. C. S., Sboeb M., Srivstava S. K., Ramana K. V. (2011). Ameliorate of experimental autoimmune uveoretinitis by aldos reductase inhibition in Lewis Tats. *Investigative Ophthalmology & Visual Science*.

[20] He C., Yu C.-R., Sun L., Mahdi R. M., Larkin J., Egwuagu C. E. (2015). Topical administration of a suppressor of cytokine signaling-1 (SOCS1) mimetic peptide inhibits ocular inflammation and mitigates ocular pathology during mouse uveitis. *Journal of Autoimmunity*.

[21] Shao H., Lei S., Sun S. L., Kaplan H. J., Sun D. (2003). Conversion of monophasic to recurrent autoimmune disease by autoreactive T cell subsets. *Journal of Immunology*.

[22] Diedrichs-Möhring M., Hoffmann C., Wildner G. (2008). Antigen-dependent monophasic or recurrent autoimmune uveitis in rats. *International Immunology*.

[23] Shao H., Shi H., Kaplan H. J., Sun D. (2005). Chronic recurrent autoimmune uveitis with progressive photoreceptor damage induced in rats by transfer of IRBP-specific T cells. *Journal of Neuroimmunology*.

[24] Yoshida Y., Yoshimi R., Yoshii H. (2014). The transcription factor IRF8 activates integrin-mediated TGF-*β* signaling and promotes neuroinflammation. *Immunity*.

[25] Takase H., Yu C.-R., Liu X., Fujimoto C., Gery I., Egwuagu C. E. (2005). Induction of suppressors of cytokine signaling (SOCS) in the retina during experimental autoimmune uveitis (EAU): potential neuroprotective role of SOCS proteins. *Journal of Neuroimmunology*.

[26] Yu C.-R., Mahdi R. M., Ebong S. (2004). Cell proliferation and STAT6 pathways are negatively regulated in T cells by STAT1 and suppressors of cytokine signaling. *Journal of Immunology*.

[27] Yu C.-R., Mahdi R. M., Ebong S., Vistica B. P., Gery I., Egwuagu C. E. (2003). Suppressor of cytokine signaling 3 regulates proliferation and activation of T-helper cells. *Journal of Biological Chemistry*.

[28] Yu C.-R., Mahdi R. R., Oh H.-M. (2011). Suppressor of cytokine signaling-1 (SOCS1) inhibits lymphocyte recruitment into the retina and protects SOCS1 transgenic rats and mice from ocular inflammation. *Investigative Ophthalmology & Visual Science*.

[29] Egwuagu C. E., Sun L., Kim S. H., Dambuza I. M. (2015). Ocular inflammatory diseases: molecular pathogenesis and immunotherapy. *Current Molecular Medicine*.

[30] Yu C.-R., Hayashi K., Lee Y. S. (2015). Suppressor of cytokine signaling 1 (SOCS1) mitigates anterior uveitis and confers protection against ocular HSV-1 infection. *Inflammation*.

